# Prevalence and risk factors for AMS: A systematic review and meta-analysis

**DOI:** 10.1371/journal.pone.0345796

**Published:** 2026-04-10

**Authors:** Xinzhu Liu, Lixia Tan, Zeng Ren, Xueyezi Bai, Shangyi Yong, Han Gao, Sang Ba, Lanzi Gongga

**Affiliations:** 1 Medical College, Xizang University, Lhasa, Xizang Autonomous Region, China; 2 Neuroscience Department, Xizang Autonomous Region People’s Hospital, Lhasa, Xizang Autonomous Region, China; 3 Pumajiangtang Branch of Langkazi County Central Hospital, Shannan, Xizang Autonomous Region, China; Weill Cornell University, UNITED STATES OF AMERICA

## Abstract

**Background:**

Acute Mountain Sickness (AMS) is a high-altitude-specific condition with variable prevalence and poorly defined risk factors. Despite growing global exposure to high-altitude environments, no systematic review has comprehensively synthesized AMS epidemiology.

**Objectives:**

To assess the prevalence of AMS and critically evaluate evidence on risk factors associated with increased susceptibility.

**Design:**

A systematic review and meta-analysis following the Meta-analysis of Observational Studies in Epidemiology (MOOSE) guidelines.

**Data sources:**

PubMed, Cochrane Library, Web of Science, CINAHL Plus, China Knowledge Resource Integrated Database (CNKI), Wanfang Database, Chinese Biomedical Database (CBM), and Weipu Database (VIP) were comprehensively searched for observational studies investigating the prevalence and risk factors of AMS from January 1, 2004 to December 31, 2024.

**Review methods:**

Original journal articles were included which met the inclusion criteria. The quality of the included studies was evaluated independently by two investigators. Meta-analysis was conducted using R software (v4.2.2), with estimates of AMS from pooled using a random-effects model.

**Results:**

Fifty-eight studies (n = 2,705) were included. The pooled AMS prevalence was 48.25% (95% CI: 42.58–53.96%), with substantial heterogeneity (I² = 82.3%). Significant risk factors included extreme altitude (>5500 m; RR = 1.89), rapid ascent (<6 h; RR = 1.60), marked heart rate increase (>20 bpm; RR = 2.35), and oxygen saturation decline (>10%; RR = 2.02). Prior AMS history (RR = 1.36) and male sex (RR = 1.15) were also associated with higher risk, while older age (>50 years) was protective (RR = 0.78).

**Conclusion:**

AMS affects nearly half of high-altitude visitors. Altitude, ascent rate, cardiopulmonary response, and prior AMS history are key risk determinants. These findings support pre-exposure risk stratification, staged ascent protocols, and real-time physiological monitoring. PROSPERO CRD42024595365.

## Introduction

Acute Mountain Sickness (AMS), a high-altitude-specific disease, arises when the body fails to acclimate promptly to hypoxia following a rapid ascent to high altitudes. Its symptoms are predominantly headaches, nausea, vomiting, dizziness, fatigue, and insomnia, and in severe cases, it can progress to High-Altitude Pulmonary Edema (HAPE) or High-Altitude Cerebral Edema (HACE) [1]. Early descriptions of high-altitude illness date back several centuries, with initial reports by Joseph Acosta [2] and later physiological studies by investigators such as Daniel Vergara Lope and Carlos Monge Medrano [3]. These early observations laid the foundation for the modern understanding of human responses to hypoxic high-altitude environments. In recent decades, the number of people traveling to high-altitude regions for tourism, research, or work has increased significantly. This trend has led to a rise in AMS cases, particularly among those without prior acclimatization. Beyond individual health impacts, AMS also places a burden on local healthcare systems and disrupts high-altitude industries such as trekking, mining, and scientific expeditions.

The reported prevalence of AMS varies widely, ranging from 5.5% [4] to 83% [5]. The observed discrepancies in the reported prevalence of acute mountain sickness (AMS) across studies can be attributed to several factors. First, a variety of diagnostic scales are employed in AMS assessment—including the Lake Louise AMS scoring system (LLS), the Hackett clinical score, the Acute Mountain Sickness–Cerebral score (AMS-C), the Clinical Functional Score (CFS), the visual analog scale (VAS), and the Chinese AMS Score (CAS) [6]. These instruments vary in their sensitivity and specificity, influencing case identification. Second, inconsistencies in study quality, sample size, and sampling methodologies contribute to variability in prevalence estimates and reduce the precision of the findings. Third, the diagnosis of AMS relies predominantly on subjective symptomatology in the absence of established biomarkers, rendering it susceptible to influences such as social support, physical exertion, and patient mood. As a result, under differing ascent conditions, individuals may exhibit varied symptomatic responses, further contributing to disparities in prevalence rates [7]. Moreover, the effects of major AMS risk factors—such as sex, age, physical fitness, smoking history, rate of ascent, and prior AMS experience—remain inconclusive and warrant further clarification through evidence-based systematic reviews [8]. To our knowledge, no comprehensive systematic review has synthesized the evidence on AMS prevalence and associated risk factors. This study aims to fill this knowledge gap by determining the overall AMS prevalence, exploring potential risk factors, and providing evidence-based recommendations to enhance AMS awareness, control, and treatment, and to guide high-altitude healthcare and tourism safety.

## Materials and methods

This review was conducted following the Meta-analysis of Observational Studies in Epidemiology (MOOSE) guidelines. A detailed study protocol is available on the PROSPERO website under the registration number CRD42024595365, register name Prevalence and risk factors for Acute mountain sickness: a systematic review and meta-analysis.

### Search strategy

A comprehensive search of the literature was performed using eight electronic databases: PubMed, the Cochrane Library, Web of Science, CINAHL Plus, China National Knowledge Infrastructure (CNKI), Wanfang Database, Chinese Biomedical Literature Database (CBM), and Weipu Database (VIP). Searches spanned from January 1, 2004 to December 31, 2024. The starting year of 2004 was selected to focus on studies conducted after the widespread adoption of the revised Lake Louise Scoring System (LLS) for diagnosing acute mountain sickness, which improved the consistency and comparability of AMS assessment across studies. Restricting the time frame also helped reduce methodological heterogeneity related to earlier diagnostic approaches.

The search strategies utilized a combination of MeSH terms and free-text keywords. The specific search string used in PubMed was:

((((((((((((Acute mountain sickness) OR (Acute mountain diseases)) OR (Acute mountain illnesses)) OR (Acute altitude sickness)) OR (Acute altitude diseases)) OR (Acute altitude illnesses)) OR (High altitude sickness)) OR (High altitude diseases)) OR (High altitude illnesses)) OR (High altitude cerebral edema)) OR (High altitude pulmonary edema)) OR (Acute high altitude disease)) AND (((((((((risk factors) OR (risk elements)) OR (risk markers)) OR (risk indicators)) OR (risk predictors)) OR (hazard factors)) OR (vulnerability factors)) OR (((((((prevalence) OR (Prevalency)) OR (Prevalence Rate)) OR (epidemiology)) OR (incidence)) OR (morbidity)))))

In addition to the electronic database searches, we manually reviewed the reference lists of the retrieved articles to identify further relevant publications and also searched grey literature. In cases of uncertainty or need for additional details, corresponding authors were contacted via email. As this study is based entirely on previously published research, it did not require Ethics Committee approval.

### Study selection

After the removal of duplicate studies, two investigators independently assessed the eligible publications by screening titles and abstracts, using the inclusion and exclusion criteria. Full-text articles were retrieved when at least one reviewer decided that an abstract was eligible for inclusion. Each publication was assessed independently by both investigators for final study inclusion. Disagreements were resolved by discussion.

No language restrictions were applied during the literature search. In addition to international databases (PubMed, Web of Science, Cochrane Library, and CINAHL Plus), several Chinese databases (CNKI, Wanfang, CBM, and VIP) were also searched to minimize retrieval bias. However, the studies ultimately included in the meta-analysis were all published in English and met the predefined eligibility criteria.

The criteria for inclusion of a study in the systematic review were as follows: (1) Observational studies (e.g., cohort, cross-sectional) or baseline data of clinical trials/randomized controlled trials reporting on AMS prevalence and risk factors; (2) Studies must involve human participants, with some exposed to high-altitude environments (usually ≥ 2500 m); (3) Studies must report AMS prevalence or its related risk factors; (4) No language restrictions were applied; (5) Only include literature with full-text accessibility, either publicly available or legally obtainable through libraries, research institutions, co-authors, etc; (6) Included studies should be published between January 1, 2004, and December 31, 2024. The exclusion criteria were as follows: (1) Non-peer-reviewed articles, conference abstracts, reviews, case reports, and animal studies are excluded; (2) Studies that don’t explicitly state participants’ altitude or have a participant altitude below 1500 m are excluded; (3) Studies not reporting AMS prevalence or risk factor data are excluded; (4) Studies with incomplete data or unextractable relevant data are excluded; (5) Studies with low methodological quality in quality assessment, such as those with obvious selection bias, information bias, or insufficient control of confounding factors, are excluded; (6) Studies published before January 1, 2004, are excluded.

### Study process

The literature screening followed the standard systematic review procedures. Two investigators independently screened the titles and abstracts of all retrieved records, followed by a full-text review of potentially eligible articles. Studies that did not meet the predefined eligibility criteria were excluded, and any disagreements between the reviewers were resolved through consultation with a third researcher.

**Fig 1:**
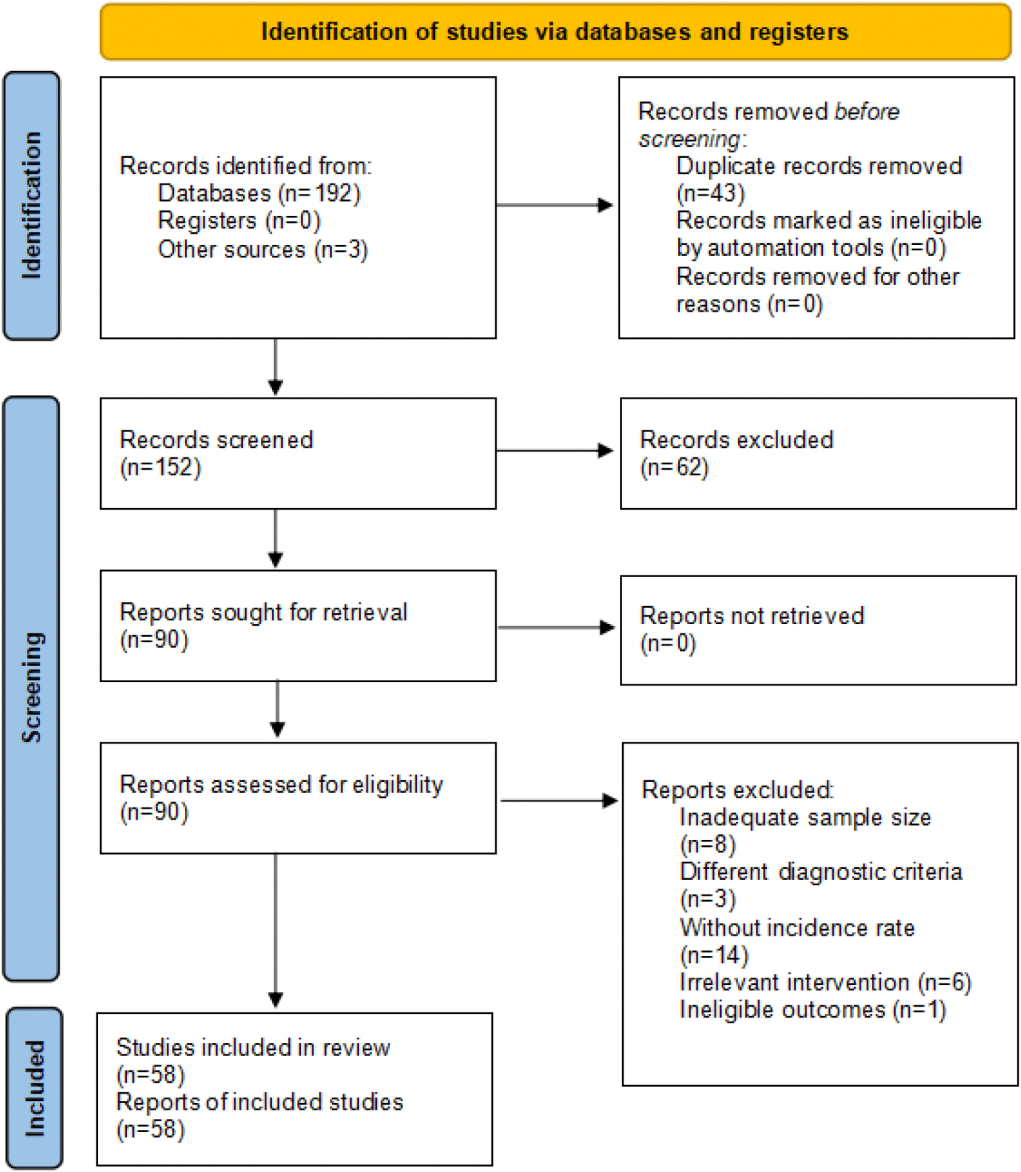
Preferred Reporting Items for Systematic Reviews and Meta-Analyses (PRISMA) flow diagram for the study selection process.

The initial search identified 195 articles, of which 43 duplicates were removed. Screening of titles and abstracts based on the inclusion criteria led to the selection of 90 articles for full-text assessment. Among these, 25 were excluded for the following reasons: inadequate sample size (n = 8), use of non-conforming diagnostic criteria (n = 3), and lack of prevalence data (n = 14). After evaluating the remaining 65 articles, six were excluded due to irrelevant interventions and one due to ineligible outcome measures. Ultimately, 58 studies involving 2,705 participants and published between 2004 and 2024 were included in the meta-analysis (Fig 1).

### Data extraction

Data were extracted from the included studies by two independent investigators. The following information was recorded: first author name, publication year, study location, sample size, diagnostic criteria, the prevalence of AMS, and stated risk factors. All extracted data were stored in the Microsoft Excel file format.

### Quality appraisal

**Fig 2:**
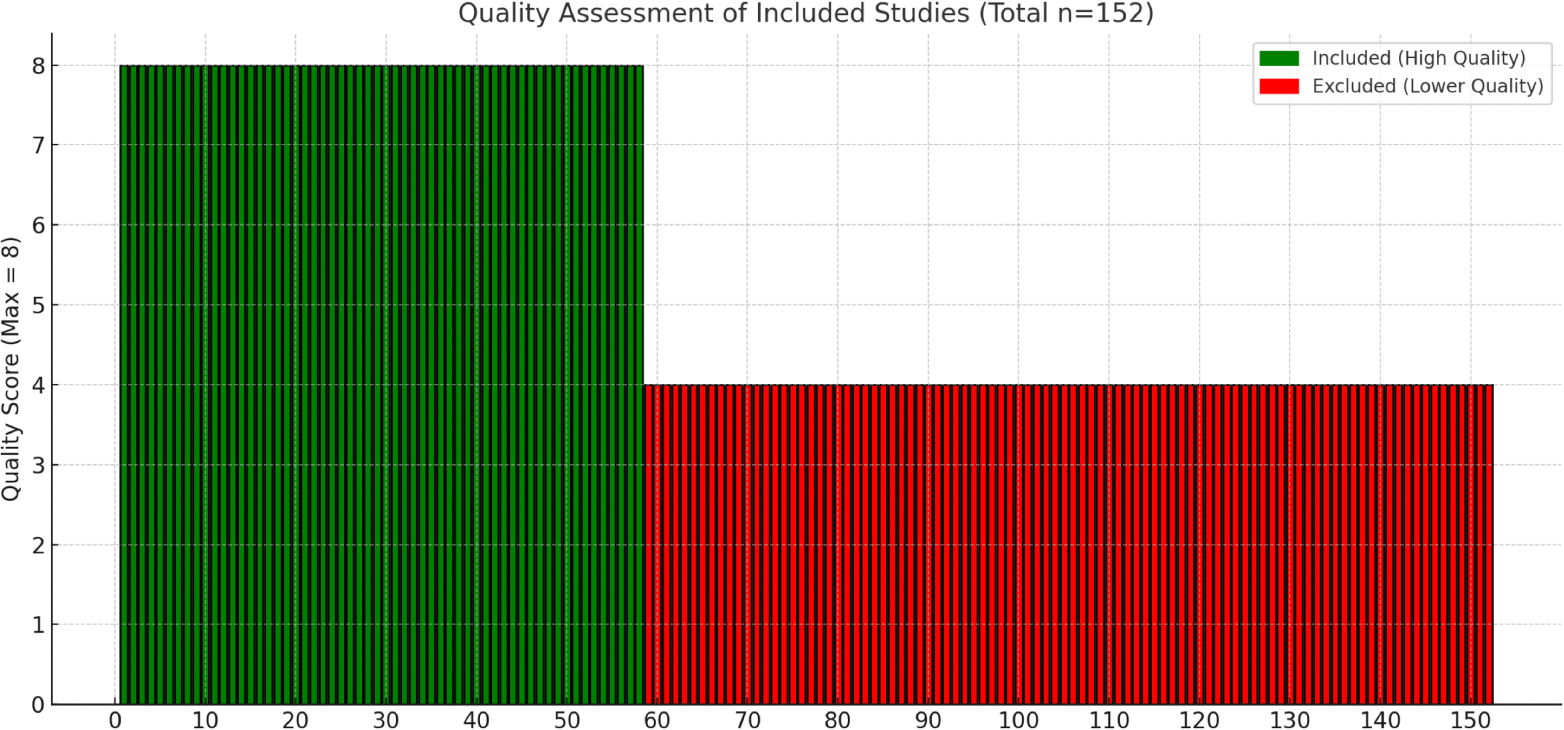
Critical appraisal of studies.

The quality of the included studies was evaluated independently by two investigators, using the tool for disease prevalence quality developed by Loney et al [9]. Any disagreement regarding the quality of studies was resolved by a third investigator. The total score of the evaluation items is 8 points. Each item that was completely compliant was scored 1, and noncompliant or partly compliant items were scored 0. A higher cumulative score indicates a smaller risk of bias in the study. As is shown in Fig 2, the greener each criterion met, the lesser the risk of bias in the studies.

### Data analysis

All statistical analyses were done with R software (v4.2.2). For AMS prevalence, we used the metaprop function in the meta package. We applied log transformation (PLOGIT) and maximum likelihood (ML) to estimate the heterogeneity parameter τ² and used a random-effects model to get the overall prevalence and 95% CI. Heterogeneity was assessed by I², with I² > 50% indicating moderate to high heterogeneity. Subgroup analyses were done to explore heterogeneity sources. For risk factor analysis, we performed meta-analysis using pooled ORs or SMDs, selecting metabin or metacont functions based on variable types. A detailed step-by-step protocol for the literature search, study selection, data extraction, quality appraisal, and statistical analysis is available at protocols.io: https://dx.doi.org/10.17504/protocols.io.dm6gpkdzjlzp/v1

## Results

### Characteristics of the included studies

The characteristics of the 58 studies are summarized in Table 1.

**Table 1:** Characteristics of included studies.

Authors	Journal/ Book	Publication Years	Diagnostic criteria	Sample size	AMS prevalence (%)	Risk factors Assessed
Small E et al. [10]	High Alt Med Biol	2021	LLQ	103	73	Sex, age, AMS history, smoking history, drinking history, altitude, diagnostic criteria, mode of entering high-altitude areas, ascent time.
Liu M et al. [11]	BMC Med	2024	LLS	50	26	Age, smoking history, drinking history, altitude, diagnostic criteria, mode of entering high-altitude areas.
Lipman GS et al. [12]	Am J Med	2018	LLQ	35	63	Sex, age, smoking history, drinking history, altitude, oxygen saturation, diagnostic criteria, mode of entering high-altitude areas, heart rate, ascent time.
Chen GZ et al. [13]	J Emerg Med	2015	LLS	20	70	Sex, age, BMI, smoking history, drinking history, altitude, oxygen saturation, diagnostic criteria, mode of entering high-altitude areas, heart rate, ascent time.
Zheng CR et al. [14]	Am J Med	2014	LLS	43	60.46	Sex, age, BMI, smoking history, drinking history, altitude, oxygen saturation, diagnostic criteria, mode of entering high-altitude areas, heart rate, ascent time.
Yang J et al. [15]	Biomed Pharmacother	2019	LLS	18	38.89	Sex, age, first entry into plateau, AMS history, smoking history, drinking history, altitude, oxygen saturation, diagnostic criteria, mode of entering high-altitude areas, heart rate, ascent time
Rexhaj E et al. [16]	Pediatrics	2011	LLS	29	62	Sex, age, BMI, first entry into plateau, AMS history, smoking history, drinking history, altitude, oxygen saturation, diagnostic criteria, mode of entering high-altitude areas, heart rate, ascent time
Wu Y et al. [17]	Travel Med Infect Dis	2023	LLS	42	28.57	Sex, age, first entry into plateau, AMS history, smoking history, drinking history, altitude, oxygen saturation, diagnostic criteria, mode of entering high-altitude areas, heart rate
Sareban M et al. [18]	Med Sci Sports Exerc	2020	AMS-C or LLS	19	11	Age, BMI, first entry into plateau, smoking history, altitude, oxygen saturation, diagnostic criteria, mode of entering high-altitude areas, heart rate, ascent time
Furian M et al. [19]	NEJM Evid	2022	AMS-C or LLS	170	32	Sex, age, BMI, first entry into plateau, smoking history, altitude, oxygen saturation, diagnostic criteria, mode of entering high-altitude areas, ascent time
Wang Y et al. [20]	CNS Neurosci Ther	2024	LLS	50	40	Age, BMI, AMS history, smoking history, drinking history, altitude, diagnostic criteria, mode of entering high-altitude areas, ascent time
Wang Z et al. [21]	Eur J Sport Sci	2022	LLS	50	40	Age, first entry into plateau, smoking history, drinking history, altitude, diagnostic criteria, mode of entering high-altitude areas, ascent time
Gatterer H et al. [22]	PLoS One	2013	LLS	43	43	Sex, age, smoking history, arterial oxygen partial pressure (PO2), partial pressure of carbon dioxide(PCO2), altitude, oxygen saturation, diagnostic criteria, mode of entering high-altitude areas, ascent time
Beidleman BA et al. [5]	High Alt Med Biol	2018	AMS-C (via ESQ)	12	83	Sex, age, first entry into plateau, smoking history, altitude, oxygen saturation, diagnostic criteria, mode of entering high-altitude areas, heart rate, ascent time
Broessner G et al. [23]	Cephalalgia	2016	LLS	77	81.18	Sex, AMS history, smoking history, altitude, diagnostic criteria, mode of entering high-altitude areas, ascent time
Schober A et al. [24]	Wilderness Environ Med	2023	LLQ	44	66.7	Sex, age, BMI, first entry into plateau, smoking history, altitude, diagnostic criteria, mode of entering high-altitude areas, ascent time
Harrison MF et al. [25]	PLoS One	2016	LLS	135	41	Sex, age, BMI, AMS history, altitude, diagnostic criteria, mode of entering high-altitude areas
Berger MM et al. [26]	J Appl Physiol (1985)	2017	LLS and AMS-C	20	35	Sex, age, BMI, smoking history, altitude, oxygen saturation, diagnostic criteria, mode of entering high-altitude areas, heart rate, ascent time
Wang X et al. [27]	Medicine (Baltimore)	2018	LLS	30	43.4	Sex, age, BMI, first entry into plateau, AMS history, smoking history, altitude, oxygen saturation, diagnostic criteria, mode of entering high-altitude areas, heart rate, ascent time
Leadbetter G et al. [28]	Wilderness Environ Med	2009	LLS and AMS-C	19	68	Sex, age, BMI, smoking history, drinking history, altitude, diagnostic criteria, mode of entering high-altitude areas, ascent time
Wille M et al. [29]	Scand J Med Sci Sports	2012	LLS	13	69.2	Sex, age, BMI, AMS history, altitude, diagnostic criteria, mode of entering high-altitude areas, heart rate, ascent time
Faulhaber M et al. [30]	Med Sci Sports Exerc	2016	LLS	15	67	Sex, age, first entry into plateau, AMS history, smoking history, drinking history, arterial oxygen partial pressure (PO2), partial pressure of carbon dioxide(PCO2), altitude, oxygen saturation, diagnostic criteria, mode of entering high-altitude areas, ascent time
Talbot NP et al. [31]	High Alt Med Biol	2011	LLS	12	67	Sex, age, first entry into plateau, AMS history, altitude, oxygen saturation, diagnostic criteria, ascent time
Jafarian S et al. [32]	Ann Neurol	2007	LLS	51	45.1	Sex, age, first entry into plateau, smoking history, altitude, diagnostic criteria, mode of entering high-altitude areas, ascent time
Mairer K et al. [33]	Int J Sports Med	2013	LLS	20	50	Sex, age, first entry into plateau, AMS history, smoking history, drinking history, altitude, oxygen saturation, diagnostic criteria, mode of entering high-altitude areas, heart rate
Iwase M et al. [34]	Echocardiography	2016	LLS	20	20	Sex, age, pulmonary artery systolic pressure (PASP), altitude, oxygen saturation, diagnostic criteria, mode of entering high-altitude areas, heart rate, ascent time
Shah N et al. [35]	High Alt Med Biol	2020	LLS	21	52.4	Sex, age, BMI, first entry into plateau, smoking history, altitude, oxygen saturation, diagnostic criteria, mode of entering high-altitude areas, heart rate, ascent time
Dumont L et al. [36]	Clin Sci (Lond)	2004	LLS	31	35.5	Sex, age, AMS history, altitude, diagnostic criteria, mode of entering high-altitude areas, ascent time
Chow T et al. [37]	Arch Intern Med	2005	LLS	20	60	Sex, age, first entry into plateau, AMS history, smoking history, altitude, diagnostic criteria, mode of entering high-altitude areas, ascent time
Schommer K et al. [38]	J Appl Physiol (1985)	2012	LLS and AMS-C	14	43	Sex, age, first entry into plateau, AMS history, smoking history, altitude, diagnostic criteria, mode of entering high-altitude areas, ascent time
Schaber M et al. [39]	Biomed Res Int	2015	LLS	37	62.2	Sex, age, BMI, first entry into plateau, AMS history, smoking history, altitude, oxygen saturation, diagnostic criteria, mode of entering high-altitude areas, heart rate, ascent time
Kanaan NC et al. [40]	J Ultrasound Med	2015	LLQ	86	55.8	Altitude, diagnostic criteria, mode of entering high-altitude areas, ascent time
Chiu TF et al. [41]	BMC Complement Altern Med	2013	LLS	102	60.8	Sex, age, BMI, first entry into plateau, AMS history, altitude, diagnostic criteria, mode of entering high-altitude areas, ascent time
Hillenbrand P et al. [4]	Wilderness Environ Med	2006	LLS	54	5.5	Sex, diagnostic criteria, mode of entering high-altitude areas, ascent time
Heo K et al. [42]	J Korean Med Sci	2014	LLS	19	74	Sex, BMI, AMS history, altitude, diagnostic criteria, mode of entering high-altitude areas, heart rate, ascent time
Gertsch JH et al. [43]	Wilderness Environ Med	2012	LLQ	109	40.4	Sex, age, AMS history, oxygen saturation, diagnostic criteria, mode of entering high-altitude areas
van Patot MC et al. [44]	High Alt Med Biol	2008	LLS and AMS-C	22	45	Sex, age, BMI, altitude, diagnostic criteria, mode of entering high-altitude areas, ascent time
Bates MG et al. [45]	High Alt Med Biol	2011	LLS	42	63	Sex, age, BMI, AMS history, pulmonary artery systolic pressure (PASP), altitude, oxygen saturation, diagnostic criteria, mode of entering high-altitude areas, heart rate, ascent time
Kayser B et al. [46]	High Alt Med Biol	2008	LLS	16	84	Age, first entry into plateau, altitude, oxygen saturation, diagnostic criteria, mode of entering high-altitude areas, heart rate, ascent time
Baillie JK et al. [47]	QJM	2009	LLS	42	66	Sex, age, BMI, first entry into plateau, AMS history, smoking history, drinking history, pulmonary artery systolic pressure (PASP), altitude, oxygen saturation, diagnostic criteria, mode of entering high-altitude areas, ascent time
Lalande S et al. [48]	Eur J Appl Physiol	2011	LLS	102	36	Sex, age, BMI, first entry into plateau, AMS history, smoking history, altitude, diagnostic criteria, mode of entering high-altitude areas, ascent time
Gatterer H et al. [49]	High Alt Med Biol	2019	LLS	13	23.1	Altitude, oxygen saturation, diagnostic criteria, mode of entering high-altitude areas, heart rate
Basnyat B et al. [50]	High Alt Med Biol	2008	LL Consensus	164	21.9	Sex, age, pulmonary artery systolic pressure (PASP), oxygen saturation, diagnostic criteria, mode of entering high-altitude areas, heart rate, ascent time
Hennis PJ et al. [51]	Nitric Oxide	2016	LLS	19	58	Sex, age, first entry into plateau, altitude, oxygen saturation, diagnostic criteria, mode of entering high-altitude areas, heart rate, ascent time
Schommer K et al. [52]	High Alt Med Biol	2010	LLS and AMS-C	20	47	Sex, age, first entry into plateau, smoking history, altitude, oxygen saturation, diagnostic criteria, mode of entering high-altitude areas, heart rate, ascent time
Wang J et al. [53]	Neurotoxicol Teratol	2013	LLS	10	50	Sex, first entry into plateau, AMS history, smoking history, altitude, diagnostic criteria, mode of entering high-altitude areas, ascent time
Moraga FA et al. [54]	Wilderness Environ Med	2007	LLQ	12	56	Sex, age, BMI, first entry into plateau, AMS history, altitude, diagnostic criteria, mode of entering high-altitude areas, heart rate, ascent time
Dehnert C et al. [55]	Wilderness Environ Med	2014	LLQ and AMS-C	21	52	Sex, age, BMI, first entry into plateau, altitude, oxygen saturation, diagnostic criteria, mode of entering high-altitude areas
Basnyat B et al. [56]	Wilderness Environ Med	2011	LLS	64	20.3	Sex, age, oxygen saturation, diagnostic criteria, mode of entering high-altitude areas, ascent time
Muza SR et al. [57]	Aviat Space Environ Med	2004	LLS	11	81.8	Sex, first entry into plateau, AMS history, altitude, diagnostic criteria
Beidleman BA et al. [58]	High Alt Med Biol	2009	LLS and AMS-C	11	72	Sex, age, first entry into plateau, AMS history, smoking history, altitude, diagnostic criteria, heart rate
Lipman GS et al. [59]	Ann Emerg Med	2012	LLQ	42	69	Sex, age, AMS history, altitude, diagnostic criteria, mode of entering high-altitude areas, ascent time
Gertsch JH et al. [60]	Wilderness Environ Med	2010	LLQ and VAS	65	28.6	Sex, age, oxygen saturation, diagnostic criteria, mode of entering high-altitude areas
Lipman GS et al. [61]	High Alt Med Biol	2015	LLQ	104	17	Sex, age, AMS history, diagnostic criteria, mode of entering high-altitude areas, ascent time
Gertsch JH et al. [62]	BMJ	2004	LLS	119	34	Sex, age, AMS history, smoking history, oxygen saturation, diagnostic criteria, mode of entering high-altitude areas
Basnyat B et al. [63]	High Alt Med Biol	2006	LLS	59	51	Sex, age, AMS history, diagnostic criteria, ascent time
Jafarian S et al. [64]	J Neurol Neurosurg Psychiatry	2008	LLS	102	47.1	Sex, age, first entry into plateau, AMS history, smoking history, drinking history, altitude, diagnostic criteria, mode of entering high-altitude areas, ascent time
Jafarian S et al. [65]	Cephalalgia	2007	LLS and VAS	12	58.3	Sex, age, AMS history, smoking history, altitude, diagnostic criteria, mode of entering high-altitude areas, ascent time

Note. LLQ = Lake Louise Questionnaire; LLS = Lake Louise AMS scoring system; AMS-C = Acute Mountain Sickness–Cerebral score; ESQ = Environmental Symptoms Questionnaire; LL Consensus = Lake Louise AMS Consensus Criteria; VAS = visual analog scale score; BMI = Body Mass Index.

### Prevalence of AMS

**Fig 3:**
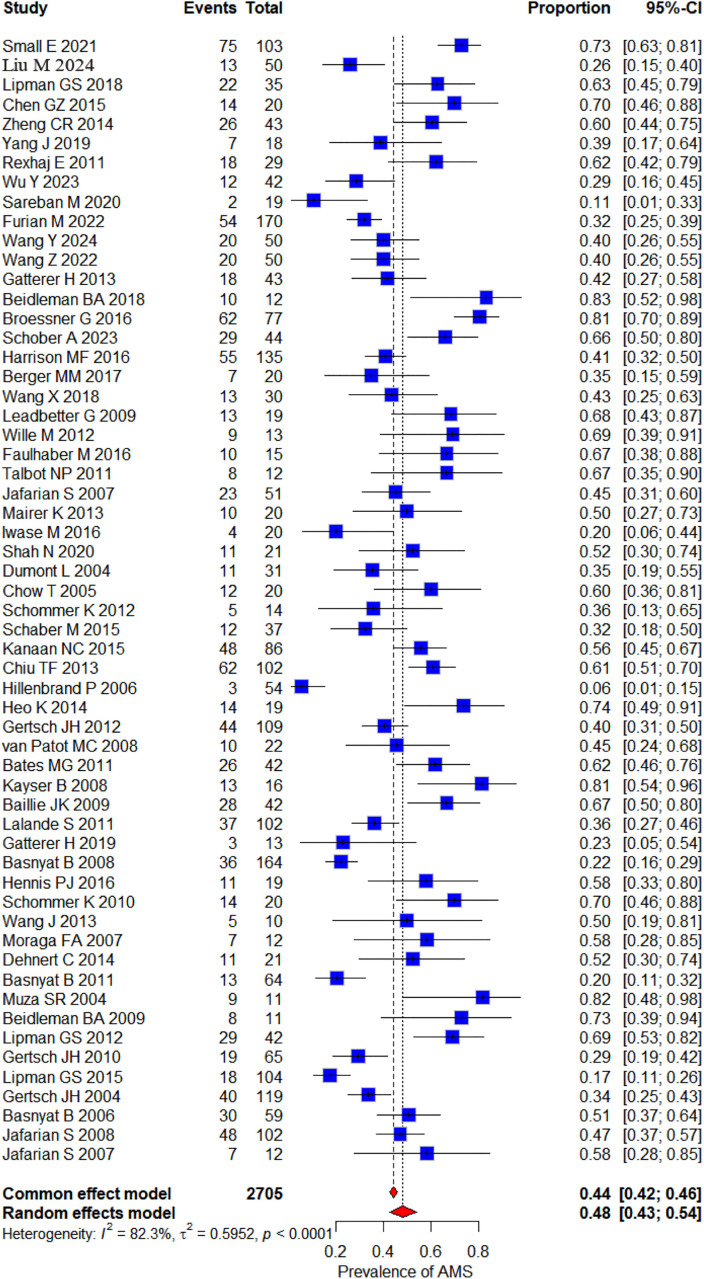
Forest plot of prevalence of AMS.

This meta-analysis included 58 studies to estimate the overall AMS prevalence. AMS prevalence across studies ranged from 5.5% [4] to 83% [5]. Using a random-effects model to pool data from all studies, the overall AMS prevalence was 48.25% (95% CI: 42.58%–53.96%). There was significant heterogeneity between studies (I² = 82.3%, P < 0.0001) (Fig 3).

### Subgroup analysis for AMS risk factors

**Fig 4:**
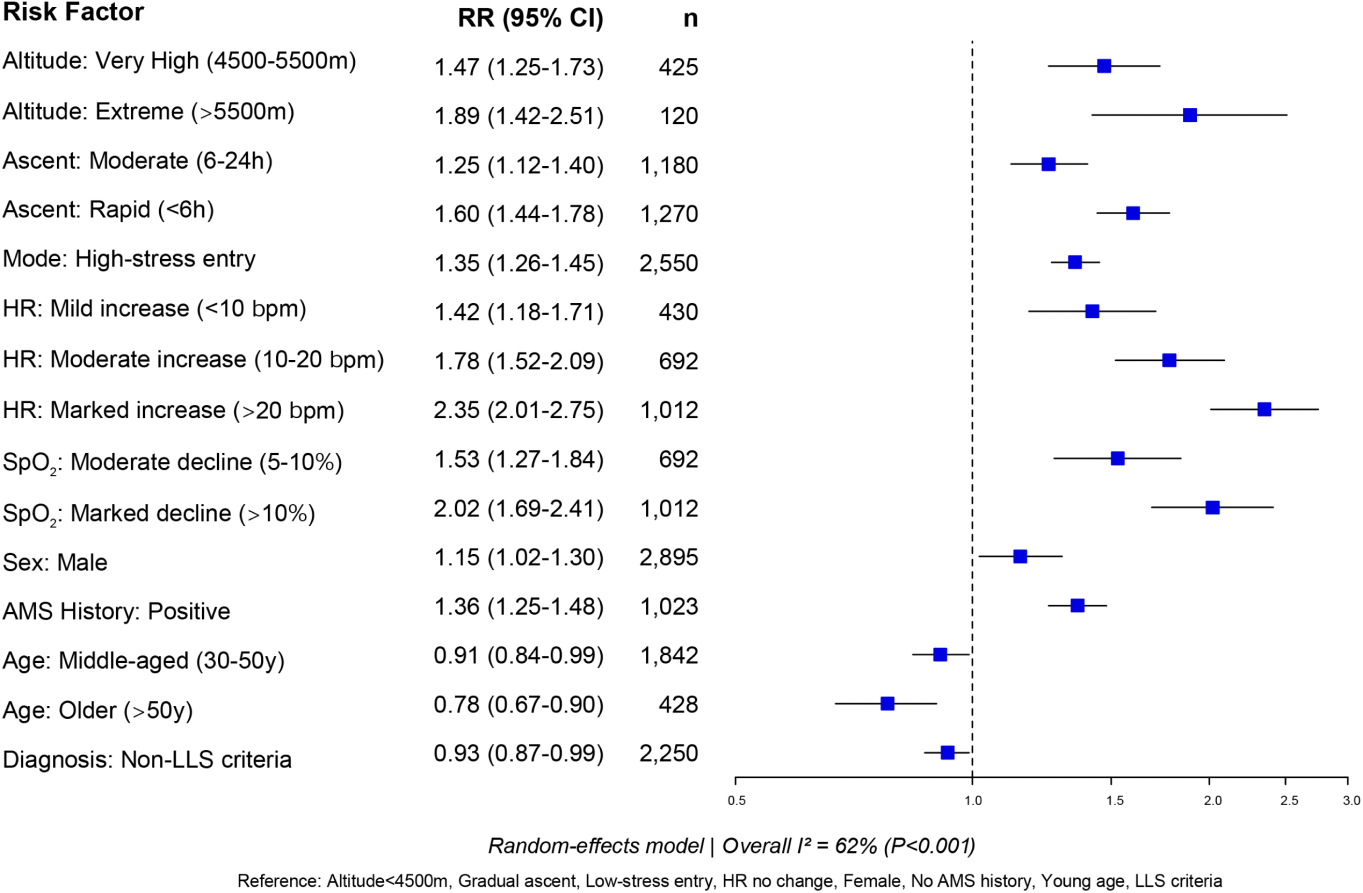
Forest plot of subgroup analysis for risk factors associated with AMS.

This subgroup analysis examined factors strongly associated with the prevalence of AMS, including target altitude, rate of ascent, stress level, heart rate change, oxygen saturation, sex, age, and history of AMS. Using an altitude below 4500 m as the reference, comparisons were made with very high altitude (4500–5500 m) and extreme altitude (>5500 m). The high-altitude group showed a significantly higher risk of AMS than the reference (RR = 1.47, 95% CI: 1.25–1.73), and the extreme-altitude group exhibited a further increased risk (RR = 1.89, 95% CI: 1.42–2.51), indicating a significant rise in AMS prevalence with ascending altitude (P < 0.05). Regarding the rate of ascent, compared with a gradual ascent, a moderate ascent (6–24 h) was associated with an increased risk of AMS (RR = 1.25, 95% CI: 1.12–1.40), and a rapid ascent was further associated with a higher risk (RR = 1.60, 95% CI: 1.44–1.78), indicating a positive correlation between ascent speed and AMS risk. An increase in heart rate was also correlated with higher AMS prevalence, with robust confidence intervals. Oxygen saturation was negatively correlated with AMS prevalence. Males had a slightly higher risk of AMS than females (RR = 1.15, 95% CI: 1.02–1.30). Participants with a history of AMS had a higher risk than those without (RR = 1.36, 95% CI: 1.25–1.48), indicating possible individual susceptibility. In terms of age, older individuals (>50 years) had a lower risk of AMS compared with younger adults (RR = 0.78, 95% CI: 0.67–0.90), suggesting that older age may be a protective factor. Additionally, the use of non-LLS diagnostic criteria was associated with a 7% lower risk of AMS compared with LLS criteria (RR = 0.93) (Fig 4).

### Quality of evidence and recommendations

**Fig 5:**
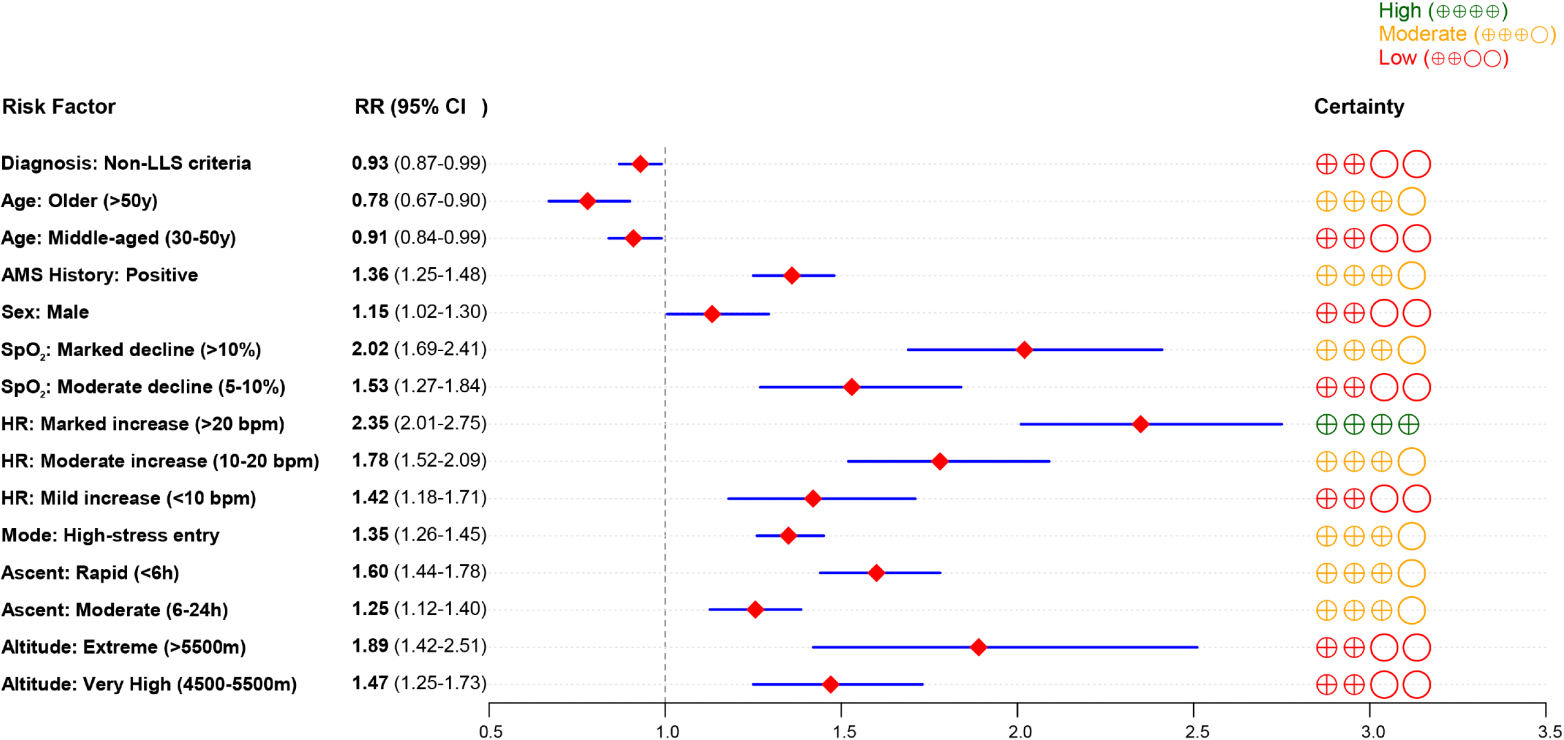
Forest Plot of Relative Risks and GRADE Certainty of Evidence for Key AMS Risk Factors.

Using the GRADE framework, we comprehensively assessed the evidence quality for 15 key risk factors associated with AMS. We detailed the combined effect size, sample size, quality of evidence, and strength of recommendation for each factor (Table 2). Among these, the evidence regarding a substantial increase in heart rate (>20 bpm) was rated as high certainty (aRR = 2.35), followed by a major reduction in blood oxygen saturation (>10%; moderate certainty, aRR = 2.02) and a rapid ascent profile (<6 h; moderate certainty, aRR = 1.60). Furthermore, we used the GRADE evidence quality forest plot (Fig 5) to visually present the relative risk (RR) of each factor and its 95% confidence interval, and mark the GRADE evidence symbols: ⨁⨁⨁⨁ (high), ⨁⨁⨁○ (moderate), ⨁⨁○○ (low). It is worth noting that extreme altitude (> 5500 m) showed the highest risk (RR = 1.89), but was downgraded to low certainty evidence due to small sample size (n = 120).

**Table 2:** GRADE Evidence Summary of Risk Factors for AMS.

Factor	Relative Risk (95% CI)	Participants (n)	Certainty	Recommendation
Altitude: Very High (4500–5500m)	1.47 (1.25–1.73)	425	Low	Conditional
Altitude: Extreme (>5500m)	1.89 (1.42–2.51)	120	Low	Conditional
Ascent: Moderate (6–24h)	1.25 (1.12–1.40)	1180	Moderate	Conditional
Ascent: Rapid (<6h)	1.60 (1.44–1.78)	1270	Moderate	Strong
Mode: High-stress entry	1.35 (1.26–1.45)	2550	Moderate	Conditional
HR: Mild increase (<10bpm)	1.42 (1.18–1.71)	430	Low	Conditional
HR: Moderate increase (10–20bpm)	1.78 (1.52–2.09)	692	Moderate	Strong
HR: Marked increase (>20bpm)	2.35 (2.01–2.75)	1012	High	Strong
SpO₂: Moderate decline (5–10%)	1.53 (1.27–1.84)	692	Low	Conditional
SpO₂: Marked decline (>10%)	2.02 (1.69–2.41)	1012	Moderate	Strong
Sex: Male	1.15 (1.02–1.30)	2895	Low	Conditional
AMS History: Positive	1.36 (1.25–1.48)	1023	Moderate	Strong
Age: Middle-aged (30–50y)	0.91 (0.84–0.99)	1842	Low	Conditional
Age: Older (>50y)	0.78 (0.67–0.90)	428	Moderate	Conditional
Diagnosis: Non-LLS criteria	0.93 (0.87–0.99)	2250	Low	Conditional

### Publication bias

**Fig 6:**
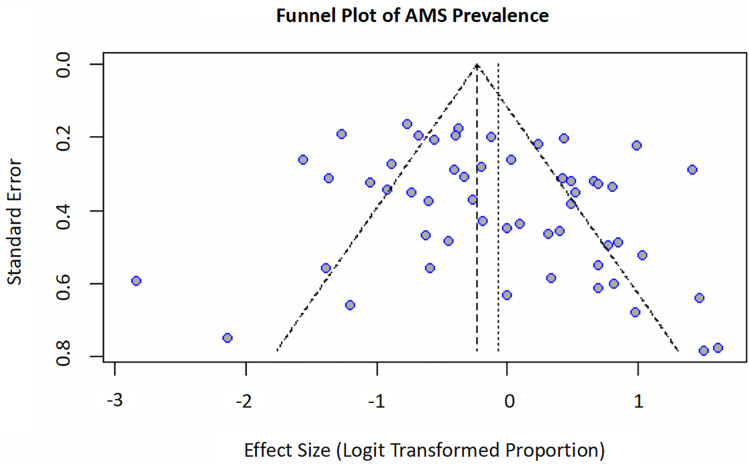
Funnel plot for assessing publication biases.

Funnel plot asymmetry (Fig 6) suggested the presence of publication bias or small-study effects. Results of Egger’s weighted regression test further supported the funnel plot asymmetry (P < 0.10).

## Discussion

This meta-analysis synthesizes evidence from 58 studies (n = 2705 participants) to elucidate the epidemiology and risk determinants of AMS. The meta-analysis yielded a pooled AMS prevalence of 48.25% (95% CI: 42.58–53.96%). Significant heterogeneity was observed across studies (I² = 82.3%), which may reflect differences in target altitude, ascent profiles, participant characteristics, diagnostic criteria, and study settings. In addition, funnel plot asymmetry and the Egger’s test result suggest the possibility of publication bias or small-study effects, which should be considered when interpreting the pooled prevalence estimate.

The estimated prevalence exhibited a broad range (5.5%–83%), highlighting the considerable influence of factors such as altitude gradient, ascent profile, and the specific diagnostic criteria applied. For example, studies conducted at extreme altitudes (>4000 m) or those involving rapid ascent protocols consistently reported higher prevalence rates, consistent with the expected increased pathophysiological stress from severe hypoxia. Furthermore, the use of different diagnostic tools (e.g., LLS versus AMS-C), which possess varying sensitivities to cerebral and systemic symptoms, constituted another major source of heterogeneity and may contribute to the over- or underestimation of the true prevalence.

At high altitude, hypoxic exposure stimulates peripheral chemoreceptors, triggering parasympathetic nervous system activation as part of the metabolic adaptation to oxygen-deficient environments [66]. This acclimatization process, however, often coincides with the development of acute mountain sickness (AMS), which, while generally self-limiting, poses a substantial health risk to high-altitude travelers. Existing epidemiological studies on AMS have typically been regional in scope. For instance, a study by Jennifer et al. involving 150 trekkers in the southern Everest region reported an AMS prevalence that increased with altitude: 0% at 2500–3000 m, 10% at 3000–4000 m, and 51% at 4000–4500 m [67]. Interestingly, prevalence declined at elevations exceeding 5000 m, a pattern potentially explained by gradual acclimatization during prolonged ascent. This observation aligns with evidence that a slower ascent rate significantly reduces AMS incidence and supports the hypothesis that mild AMS may represent part of the spectrum of physiological acclimatization—though this interpretation requires further validation. The comparatively low prevalence reported in their study relative to the pooled estimate from our meta-analysis may be attributed to the cohort’s composition of experienced trekkers and their controlled, moderate ascent profile.

Subgroup analyses revealed that exposure to extreme altitude (>5000 m) was associated with an 89% increase in AMS risk relative to baseline, establishing it as the most significant risk factor. At 5000 meters, the partial pressure of atmospheric oxygen drops to approximately half of the sea-level value [68], inducing severe hypoxemia. In response, the organism enhances adrenaline release and catecholamine secretion to maintain cardiac output, leading to markedly elevated heart rates through stimulated myocardial contraction. This meta-analysis corroborates that under acute hypoxia, variations in oxygen saturation—resulting from hypoxemia—and adrenaline-mediated increases in heart rate exhibit an inverse relationship. Both parameters demonstrate a bidirectional association with AMS incidence, functioning not only as predisposing factors for its onset but also as exacerbating elements after AMS manifestation.

Notably, age appeared to confer a protective effect against AMS. Compared with the younger reference group, middle-aged and older participants showed 9% and 22% reductions in AMS risk, respectively. This pattern may be linked to the functional responsiveness of carotid body-adrenomedullary arterial chemoreceptors, which serve as central sensors for blood oxygen levels and initiate cardiopulmonary reflexes—such as hyperventilation and sympathetic activation—upon detecting hypoxia to promote acclimatization [69]. An alternative mechanistic explanation is that age-dependent cerebral atrophy enlarges the intracranial buffering compartment, thereby accommodating mild vasogenic oedema without a clinically relevant rise in intracranial pressure and, consequently, attenuating either the incidence or the severity of AMS [70]. Further investigation into the mechanistic basis of this age-dependent protection may yield novel strategies for AMS prevention.

Additionally, male gender was associated with a moderately elevated AMS risk compared with female participants (RR = 1.15). Collectively, these findings underscore the importance of managing modifiable environmental exposures—particularly altitude and ascent rate—as controllable risk factors, while reinforcing the value of real-time monitoring of heart rate and oxygen saturation as essential components of AMS risk mitigation.

Several methodological limitations should be considered when interpreting our findings. First, the reliance on symptom-based diagnostic criteria introduces an element of subjectivity, as symptom reporting may be influenced by psychological state (e.g., anxiety) or cultural perceptions of illness. Second, although no language restrictions were applied during the literature search and both international and Chinese databases were examined, the studies ultimately included in the meta-analysis were all published in English. Therefore, the possibility of language bias cannot be completely excluded. In addition, publication bias may also exist, as suggested by funnel plot asymmetry and Egger’s test (P < 0.10). Most importantly, the current approach to AMS diagnosis lacks objective biomarker support. The absence of physiological or biochemical markers hinders the identification of subclinical AMS cases and complicates the differential diagnosis from other high-altitude conditions with overlapping symptomatology [71]. Future studies incorporating validated biomarkers are needed to improve diagnostic accuracy and pathological understanding.

From a clinical perspective, these findings support the implementation of pre-ascent risk stratification, with particular attention to individuals with a history of AMS or suboptimal physiological indicators. Public health efforts could benefit from the widespread adoption of standardized ascent guidelines—such as staged ascent with a daily gain not exceeding 300 m above 2,500 m—along with the promotion of real-time blood oxygen saturation monitoring to reduce AMS incidence [72]. In the research domain, future prospective studies that integrate genomic, metabolic, and environmental data are warranted to identify predictive biomarkers and improve risk prediction models [73]. Furthermore, context-specific interventions—including altitude pre-acclimatization protocols or pharmacological prophylaxis (e.g., acetazolamide)—should be evaluated across diverse populations to enhance generalizability and clinical applicability [62].

This meta-analysis synthesizes previously fragmented evidence into a consolidated risk profile for AMS. The results offer a basis for refining pre-travel health assessments, optimizing ascent strategies, and assisting clinicians in the early identification of high-risk individuals prior to symptom onset.

## Conclusion

This systematic review and meta-analysis, synthesizing evidence from 58 studies involving 2,705 participants, confirms that AMS remains highly prevalent among high-altitude travelers, with a pooled prevalence of 48.25%. The findings establish extreme altitude exposure (>5500 m), rapid ascent profiles (<6 hours), pronounced cardiopulmonary responses (heart rate increase >20 bpm; oxygen saturation decline >10%), and previous AMS episodes as consistent risk determinants. Considerable heterogeneity observed across studies highlights methodological variations in diagnostic approaches and ascent conditions. These results support the implementation of staged ascent protocols, pre-travel risk assessment targeting susceptible individuals, and real-time physiological monitoring as effective strategies for reducing the global burden of AMS.

## Supporting information

S1 File PRISMA_2020_checklist for this systematic review and meta-analysis. (DOCX)
